# Molecular Epidemiology of *Staphylococcus aureus* Bacteremia: Association of Molecular Factors With the Source of Infection

**DOI:** 10.3389/fmicb.2018.02210

**Published:** 2018-09-25

**Authors:** Dafne Pérez-Montarelo, Esther Viedma, Nieves Larrosa, Carmen Gómez-González, Enrique Ruiz de Gopegui, Irene Muñoz-Gallego, Rafael San Juan, Nuria Fernández-Hidalgo, Benito Almirante, Fernando Chaves

**Affiliations:** ^1^Hospital Universitario 12 de Octubre, Madrid, Spain; ^2^Hospital Universitario Vall d'Hebron, Barcelona, Spain; ^3^Hospital Galdakao-Usansolo, Galdakao, Spain; ^4^Hospital Universitario Son Espases, Palma de Mallorca, Spain

**Keywords:** *Staphylococcus aureus* bacteremia, bacteremia source, molecular epidemiology, clonal complex, virulence factors

## Abstract

*Staphylococcus aureus* bacteremia (SAB) is associated with high morbidity and mortality, which varies depending on the source of infection. Nevertheless, the global molecular epidemiology of SAB and its possible association with specific virulence factors remains unclear. Using DNA microarrays, a total of 833 *S. aureus* strains (785 SAB and 48 colonizing strains) collected in Spain over a period of 15 years (2002–2017) were characterized to determine clonal complex (CC), *agr* type and repertoire of resistance and virulence genes in order to provide an epidemiological overview of CCs causing bloodstream infection, and to analyze possible associations between virulence genes and the most common sources of bacteremia. The results were also analyzed by acquisition (healthcare-associated [HA] and community-acquired [CA]), methicillin-resistant (MRSA) and methicillin-susceptible (MSSA) strains, and patient age (adults vs. children). Our results revealed high clonal diversity among SAB strains with up to 28 different CCs. The most prevalent CCs were CC5 (30.8%), CC30 (20.3%), CC45 (8.3%), CC8 (8.4%), CC15 (7.5%), and CC22 (5.9%), which together accounted for 80% of all cases. A higher proportion of CC5 was found among HA strains than CA strains (35.6 vs. 20.2%, *p* < 0.001). CC5 was associated with methicillin resistance (14.7 vs. 79.4%, *p* < 0.001), whereas CC30, CC45, and CC15 were correlated with MSSA strains (*p* < 0.001). Pathogen-related molecular markers significantly associated with a specific source of bacteremia included the presence of *sea, undisrupted hlb* and *isaB* genes with catheter-related bacteremia; *sed, splE*, and *fib* genes with endocarditis; *undisrupted hlb* with skin and soft tissue infections; and finally, CC5, *msrA* resistance gene and *hla* gene with osteoarticular source. Our study suggests an association between *S. aureus* genotype and place of acquisition, methicillin resistance and sources of bloodstream infection, and provides a valuable starting point for further research insights into intrinsic pathogenic mechanisms involved in the development of SAB.

## Introduction

*Staphylococcus aureus* is an opportunistic pathogen that can potentially cause a wide range of infections. It is a leading cause of bacteremia and represents a significant global health problem (Weiner et al., 2016). *S. aureus* bacteremia (SAB) is often associated with severe metastatic infections, such as infective endocarditis, septic arthritis and osteomyelitis and complications, such as sepsis and septic shock, which lead to adverse outcomes that are challenging to manage (Shorr et al., 2006; Wyllie, 2006).

The incidence of SAB is difficult to determine and there are major geographical differences that reflect discrepancies in health care systems and infection control practices. In developed countries, the estimated incidence 80–190 cases per 100,000 inhabitants per year (Laupland, 2013; Le Moing et al., 2015). Despite the improvements in SAB management, including greater understanding of this infection and mandatory surveillance implemented in several countries over recent decades, SAB still causes significant morbidity and mortality, with an associated early mortality that appears to have plateaued at approximately 20–30% (van Hal et al., 2012). Certainly, little is known about global SAB epidemiology in terms of the circulating clones causing SAB in different patient subgroups, such as adults and children, or those most commonly found in the community or hospital settings. Because it is becoming progressively more difficult to differentiate between healthcare-associated and community-acquired infections due to changes in the complexity of present health care systems, it is important to identify the specific clones that are traditionally associated with the community but may be entering hospitals and replacing common nosocomial clones, and vice versa. Moreover, it would be especially interesting to study clonality taking into account that bacterial phenotype and genotype have been shown to have a possible influence on infection outcome, since different clones can adopt different strategies to overcome host responses and cause severe pathology (Recker et al., 2017). The overall mortality rate from SAB varies depending on the primary focus of infection (the highest rates occur in patients with infective endocarditis and pulmonary infections, and the lowest in patients with catheter-related infections) and on the complications deriving from SAB. This association makes it necessary to regard SAB not as a single entity, but as a heterogeneous group of infections that can evolve differently and therefore require source-specific management (van Hal et al., 2012). However, the characteristics of the most common clones causing SAB according to source of infection remain unknown. Furthermore, determining the role of particular genetic backgrounds (clonality and virulence) in bloodstream infections caused by *S. aureus* has become a real challenge due to the diversity, redundancy and host specificity of the virulence factors.

The aim of the present study was to explore the molecular characteristics of *S. aureus* strains causing bacteremia in order to provide an epidemiological overview of the circulating clones causing bloodstream infection and to analyze the possible association between virulence and the most common sources of bacteremia.

## Materials and methods

### Data collection

A total of 785 strains causing bacteremia with different source and 48 colonization strains collected over a period of 15 years (2002–2017) were analyzed. These strains were obtained from different sources in hospitals geographically distant from each other spread across the territory of Spain (Table 1). Specifically, these strains were identified in 10 different collections: six were single-center studies developed at the Hospital 12 de Octubre in Madrid, and the remaining four corresponded to multi-center studies developed at various Spanish hospitals (Muñoz-Gallego et al., 2017; San-Juan et al., 2017; Fernández-Hidalgo et al., 2018). The main focus and objective of the studies for which these strains were collected was source of staphylococcal bacteremia, mainly endocarditis (*N* = 214), catheter-related bacteremia (CRB) (*N* = 212), skin and soft tissue infections (SSTI) (*N* = 66), and bone and joint infections (*N* = 100). Eight of these collections corresponded to SAB infections in adults, and two in children (< 15 years of age). The percentage of MRSA strains included in each collection varied. The studies were approved by the ethics committee of the University Hospital 12 de Octubre (Madrid, Spain). It was not considered necessary to obtain written informed consent because the participants were anonymized (IRH-ANT-2013-01).

**Table 1:** Description of the bacteremia-producing strain collections included in the study.

**Source**	**Period**	**Participating cities**	Participating hospitals^*^	**Number of strains**	**Age population**	**MRSA (%)**	**Commentary**	**References**
Endocarditis	2013–2016	Malaga, Seville, Madrid, Barcelona, Santander, Bilbao	15	210	Adults	19.0	Prospective study	Fernández-Hidalgo et al., 2018
Catheter	2011–2014	Madrid, Barcelona, Seville	5	80	Adults	0.0	Prospective study including only MSSA strains	San-Juan et al., 2016
Various sources^a^	2002–2010	Madrid	1	111	Children	3.6	Retrospective studies	–
Various sources^b^	2009–2010	Madrid, Barcelona, Palma de Mallorca, A Coruña	5	91	Children	12.1	Prospective study	–
Various sources^b^	2006–2010	Madrid	1	45	Adults and children	51.1	Retrospective study including recurrent bacteremia. Children *n* = 3	–
Various sources^d^	2010–2011	Madrid	1	111	Adults	24.3	Retrospective study	Viedma et al., 2014
Various sources^e^	2012–2014	Madrid	1	59	Adults	100.0	Retrospective study including only MRSA strains	–
Bone and joint infections	2005–2015	Madrid	1	49	Adults	71, 4	Retrospective study	Muñoz-Gallego et al., 2017
Bone and joint infections	2016	Madrid	7	29	Adults	20, 7	Prospective study	–
Colonization	2012	Madrid	1	48	Adults	5.8	Prospective study of colonization strains from healthy carriers	López-aguilera et al., 2013

a *Catheter-related, congenital, skin and soft tissue infections, osteoarticular, unknown*.

b, c, e *Catheter-related, skin and soft tissue infections, osteoarticular, unknown*.

d *Catheter-related, skin and soft tissue infections, osteoarticular, endocarditis, urinary tract infection, respiratory, unknown*.

* *Participant Hospitals: University Hospital 12 de Octubre, University Hospital de La Princesa, University Hospital Puerta del Hierro, University Hospital La Paz (Comunidad de Madrid); University Hospital A Coruña (Galicia); University Hospital Marqués de Valdecilla (Cantabria); University Hospital Son Espases (Islas Baleares); University Hospital Cruces (País Vasco); University Hospital Vall d‘Hebron, Hospital Universitario de Bellvitge, Hospital de la Santa Creu y Sant Pau, Hospital de San Pedro, Hospital Germans Trias i Pujol, Hospital de Barcelona, Hospital Parc Tauli (Cataluña); Hospital Universitario Virgen de la Macarena, Hospital Universitario Virgen del Rocío, Hospital Universitario Virgen de la Victoria (Andalucía)*.

Cases were classified according to acquisition: healthcare-associated (HA) or community-acquired (CA). HA included both nosocomial cases with a positive blood culture obtained from patients who had been hospitalized for 48 h or longer (Garner et al., 1988) and healthcare-associated cases following Friedman et al.'s criteria (Friedman et al., 2002). CA cases were those with a positive blood culture obtained at the time of hospital admission or within 48 h after hospital admission.

Methicillin resistance was defined on the basis of results of microdilution techniques, cefoxitin susceptibility testing and/or the presence of the *mecA* gene.

### Molecular studies

Blood cultures were processed with an automated blood culture system (BACTEC 9240, Becton Dickinson Microbiological System, USA). Automatic microdilution techniques were used for identification and susceptibility testing of isolates. Bacterial DNA was extracted using commercial extraction kits (Qiagen, Germany) according to the manufacturer's recommendations. DNA microarrays (Alere, Germany; Monecke et al., 2008) covering 334 target sequences and approximately 187 different genes that included species-specific markers, antimicrobial resistance genes, exotoxins, genes encoding microbial surface components recognizing adhesive matrix molecules (MCSCRAMMs), capsule genes, clonal complexes (CC) and *agr* group typing markers were run on the whole collection of strains. Those cases with ambiguous array results were considered as missing values for further analysis.

Only genes found with a frequency of between 5 and 95% in the whole collection were considered for statistical analysis.

### Statistical analysis

Categorical variables were compared using the chi-squared or Fisher's exact test, as appropriate. Significance levels of DNA microarray results were corrected using the Bonferroni correction for multiple tests. Pairwise comparisons of the main CCs, *agr* types and virulence genes were performed with source of bacteremia. Potential associations were investigated by univariate and multivariate logistic regression, in which CCs, *agr* types and virulence factors were considered as independent dichotomous variables, and source of bacteremia as the dependent variable. For multivariate analysis, variables with a *p*-value <0.1 in the univariate analysis were included in a backward stepwise algorithm. All statistical tests were two-tailed and a *p*-value of <0.05 was considered statistically significant. Analyses were performed using the SPSS statistical package, version 21.0 (SPSS Inc., Chicago, IL).

## Results

A total of 785 *S. aureus* strains causing bacteremia and 48 *S. aureus* colonizing strains were characterized in this study with the DNA microarray. Of a total of 187 genes included in the array, 67 genes were excluded from further analysis. Twenty one genes because they were found in almost all of the strains (>95%): *sarA*(99.5%)*, saeS*(98.4%)*, vraS*(99.7%)*, lukF*(96.7%)*, hl*(97.5%)*, hld*(99.3%)*, sspA*(99.8%)*, sspB*(100%)*, sspP*(99.8%)*, icaA*(99.6%)*, icaC*(98.4%)*, icaD*(99.5%)*, clfA*(99.6%)*, clfB*(99.4%)*, ebpS*(99.4%)*, eno*(99.5%)*, fnbA*(98.1%)*, map*(97.6%)*, sdrC*(99.3%) and *isdA*(99.6%); and 47 genes because they were absent from almost all of the strains (< 5%): *ermA*(4.7%)*, ermB*(0.3%)*, lnuA*(0.7%)*, mefA*(0%)*, vatA*(0%)*, vatB*(0%)*, vgaA*(2.6%)*, vgaB*(0%)*, aacA-aphD*(3.9%)*, dfrS1*(2.5%)*, fusB*(0%)*, fusC*(0.8%)*, tetK*(2.2%)*, tetM*(2.1%)*, cat*(0.8%)*, cfr*(0.1%)*, fexA*(0.2%)*, qacA*(0.2%)*, qacC*(2.2%)*, vanA*(0%)*, vanB*(0%)*, vanZ*(0%)*, seb*(3.6%)*, see*(0%)*, seh*(4.6%)*, sej*(3.6%)*, sek*(3.4%)*, seq*(3.1%)*, ser*(3.4%)*, pvl*(1.8%), *etA*(2.5%)*, etB*(0.8%)*, etD*(3.1%)*, edinA*(0.6%)*, edinB*(3.3%)*, edinC*(0.7%)*, ACME cluster*(0.5%)*, arcA-SCC*(0.2%)*, arcB-SCC*(0.1%)*, arc-SCC*(0.1%)*, arcD-SCC*(3.6%)*, cap1*(0.7%), and *bap*(0.1%). Nine additional genes were discarded due to an unacceptable number of missing values (>30%): *mecC*(55%), *mecR*(56.2%)*, merA*(53.3%)*, merB*(39.5%)*, fosBplasmid*(62.8%)*, lukM*(52.8%)*, lukY*(51.3%)*, setC-selX*(52.3%)*, setB3*(51.5%)*, setB2*(51.7%)*, setB1*(52.6%)*, fnbB*(51.3%), and *sdrD*(51.6%).

The main CCs detected in this study were: CC5 (30.8%), CC30 (20.3%), CC45 (8.3%), CC8 (8.4%), CC15 (7.5%) and CC22 (5.9%). In addition, up to 22 minor CCs were also detected: CC398 (2.4%), CC121 (2.4%), CC25 (2.2%), CC9 (1.7%), CC97 (1.6%), CC6 (1.3%), CC1 (1.1%), CC7 (1.1%), CC188 (1.0%), CC101, CC10, CC49, CC59, CC509, CC20, CC12, CC75, CC96, CC395, CC522, CC707 and CC1021 (<1%; Table 2). *agrII* type was the most common (41.7%), followed by *agrI* (33.7%) and *agrIII* (22.2%).

**Table 2:** Distribution of *S. aureus* clonal complexes according to place of acquisition, methicillin resistance and age of population.

	Place of acquisition^*^			**Methicillin resistance**			**Age (adult)**		
CC^**^	**HA** ***N*** = **551** ***n*** **(%)**	**CA** ***N*** = **255** ***n*** **(%)**	***P*****-value**	**MSSA** ***N*** = **627** ***n*** **(%)**	**MRSA** ***N*** = **206** ***n*** **(%)**	***P*****-value**	**Adult** ***N*** = **628** ***n*** **(%)**	**Children** ***N*** = **205** ***n*** **(%)**	***P*****-value**
5	193 (35.6)	50 (20.2)	<**0.001**	90 (14.7)	162 (79.4)	<**0.001**	213 (34.2)	39 (20.0)	<**0.001**
8	38 (7.0)	21 (8.5)	0.474	42 (6.9)	20 (9.8)	0.179	45 (7.2)	17 (8.7)	0.501
15	40 (7.4)	21 (8.5)	0.598	61 (10.0)	0 (0.0)	<**0.001**	43 (6.9)	18 (9.2)	0.293
22	35 (6.5)	11 (4.4)	0.249	39 (6.4)	9 (4.4)	0.291	39 (6.3)	9 (4.6)	0.379
30	101 (18.6)	58 (23.4)	0.126	165 (26.9)	1 (0.5)	<**0.001**	113 (18.2)	53 (27.2)	**0.008**
45	42 (7.7)	23 (9.3)	0.473	65 (10.6)	4 (2.0)	<**0.001**	50 (8.0)	19 (9.7)	0.462

* *27 strains did not have place of acquisition data available*.

** *Only the major clones are shown. Other clones detected in this study were: CC1(1.1%), CC6(1.3%), CC7(1.1%), CC9(1.7%), CC10(0.6%), CC12(0.2%), CC20(0.4%), CC25(2.2%), CC49(0.6%), CC59(0.6%), CC75(0.1%), CC96(0.1%), CC97(1.6%), CC101(0.7%), CC121(2.4%), CC188(1.0%), CC395(0.1%), CC398(2.4%), CC509(0.5%), CC522(0.1%), CC707(0.1%) and CC1021(0.1%)*.

*Statistically significant results are highlighted in bold*.

*CC, clonal complex; HA, healthcare-associated; CA, community-associated; MSSA, methicillin-susceptible S. aureus; MRSA, methicillin-resistant S. aureus*.

### Distribution of *S. aureus* strains by CC and *agr* type according to acquisition, methicillin resistance and age of population

#### Healthcare-associated vs. community-acquired

In our collection, there was a higher proportion of HA strains (68.4%) compared to CA strains, which accounted for 31.6%. Healthcare-associated strains were assigned to 26 different CCs, with CC5 (35.6%) being the most common, followed by CC30 (18.6%). The CC diversity was slightly lower among CA strains, which were assigned to 23 CCs, with CC30 (23.4%) and CC5 (20.2%) being the ones most commonly found (Table 2). Although most clones circulated in the healthcare and community settings, a higher proportion of CC5 was found among HA than among CA strains (35.6 vs. 20.2%, *p* < 0.001; Table 2).

The distribution of *agr* types is presented in Table 3. Note that while *agrI* was the main *agr* type among CA strains (31.0% vs. 39.2%, *p*: 0.030), *agrII* was associated with HA acquisition, (46.6 vs. 31.8%, *p* < 0.001).

**Table 3:** Distribution of *agr* types according to place of acquisition, methicillin resistance and age of population.

	Place of acquisition^*^			**Methicillin resistance**			**Adult age**		
***agr*** type^**^	**HA** ***N*** = **551** ***n*** **(%)**	**CA** ***N*** = **255** ***n*** **(%)**	***P*****-value**	**MSSA** ***N*** = **627** ***n*** **(%)**	**MRSA** ***N*** = **206** ***n*** **(%)**	***P*****-value**	**Adult** ***N*** = **628** ***n*** **(%)**	**Children** ***N*** = **205** ***n*** **(%)**	***P*****-value**
*agrI*	169 (31.0)	96 (39.2)	**0.030**	238 (38.6)	37 (18.4)	<**0.001**	206 (33.3)	69 (34.7)	0.728
*agrII*	254 (46.6)	78 (31.8)	<**0.001**	179 (29.1)	162 (80.6)	<**0.001**	276 (44.7)	65 (32.7)	<**0.001**
*agrIII*	113 (20.7)	61 (24.9)	0.225	179 (29.1)	2 (1.0)	<**0.001**	123 (19.9)	58 (29.1)	**0.008**
*agrIV*	9 (1.7)	10 (4.1)	0.070	20 (3.2)	0 (0.0)	**0.006**	13 (2.1)	7 (3.5)	0.390

* *27 strains did not have data for place of acquisition available*.

** *In the whole collection there were 16 non-typable agr strains*.

*Statistically significant results are highlighted in bold*.

*HA, healthcare-associated; CA, community-associated; MSSA, methicillin-susceptible S. aureus; MRSA, methicillin-resistant S. aureus*.

#### Methicillin resistance

Methicillin resistance was observed in 24.7% of the strains. Eleven different CCs were detected in this group, with CC5 being the main clone (79.4%). By contrast, among the MSSA strains, all the CCs detected in our collection were represented (*N* = 28) and showed a higher clonal diversity than MRSA strains. The major clones detected among MSSA strains were CC30 (26.5%), CC5 (14.5%), CC45 (10.5%) and CC15 (10.0%). A comparison of the two groups revealed that only CC5 was associated with methicillin resistance (14.7 vs. 79.4%, *p* < 0.001), whereas CC30, CC45 and CC15 were correlated with MSSA strains (*p* < 0.001; Table 2).

With respect to *agr* type, *agrII* type was observed to be significantly associated with MRSA strains (29.1 vs. 80.6%, *p* < 0.001), whereas, *agrI* was associated with MSSA strains (38.6 vs. 18.4%, *p* < 0.001; Table 3).

#### Adult vs. child population

While 28 CCs detected in this study were represented among strains isolated from adults, lower CC diversity was detected in children, with only 19 CCs identified. A comparison of clonality in the adult and children populations revealed that while CC5 was associated with strains from adults (34.2 vs. 20.0%, *p* < 0.001), CC30 was significantly related to strains from the child population (18.2 vs. 27.2%, *p* < 0.008; Table 2).

The distribution of *agr* types by patient age is shown in Table 3. This analysis showed a significant association between *agrII* and strains from adults (44.7 vs. 32.7%, *p* < 0.001), while *agrIII* was associated with the child population (19.9 vs. 29.1%, *p* < 0.008).

### Clonal complex diversity, *agr* type and virulence genes among *S. aureus* strains from different sources of bacteremia

The main objective was to explore the distribution of CCs and virulence genes according to source of bacteremia. A collection of *S. aureus* strains from healthy carriers was also added to the analysis in order to evaluate potential differences between colonizing and bacteremic strains.

Remarkably, CC5 and *agrII* predominated in SAB from osteoarticular infections (Tables 4A,B). When we focused on antibiotic resistance genes, significant differences were identified in strains from different sources of infection for the *mecA, msrA, aadD, aphA3*, and *sat* genes (Tables 4A,B). Our results seemed to indicate a higher proportion of these resistance genes among osteoarticular infections compared with other bacteremia sources. In general, significant differences for source of bacteremia and colonization were also detected in virulence genes, such as *sea, sed, hla, undisrupted hlb, splE, cna, fib*, and *isaB* among others.

**Table 4A:** Frequency of clonal complexes, *agr* type, resistance and virulence genes according to different sources of infection.

**Variable**	**Colonization (*N* = 48) *n* (%)**	**CRB (*N* = 212) *n* (%)**	**Endocarditis (*N* = 214) *n* (%)**	**SSTI (*N* = 66) *n* (%)**	**Osteoarticular (*N* = 100) *n* (%)**	***P*-value^a^**
CC^*^						
5	9 (19.1)	65 (31.3)	47 (22.0)	19 (29.7)	51 (51.5)	<**0.001**
8	2 (4.3)	14 (6.7)	22 (10.3)	5 (7.8)	3 (3.0)	0.236
15	3 (6.4)	15 (7.2)	20(9.3)	10 (15.6)	4 (4.0)	0.192
22	2 (4.3)	15 (7.2)	16 (7.5)	1 (1.6)	7 (7.1)	0.435
30	11 (23.4)	44 (21.2)	41 (19.2)	11 (17.2)	11 (11.1)	0.263
45	4 (8.5)	19 (9.1)	19 (8.9)	3 (4.7)	6 (6.1)	0.736
***agr*** **TYPES**						
*agrI*	17 (36.2)	72 (34.1)	81 (38.6)	18 (28.1)	28 (28.3)	0.342
*agrII*	14 (29.8)	90 (42.7)	77 (36.7)	31 (48.4)	55 (55.6)	0.008
*agrIII*	12(25.5)	47 (22.3)	46 (21.9)	14 (21.9)	12 (12.1)	0.184
*agrIV*	4 (8.5)	2 (0.9)	6 (2.9)	1 (1.6)	4 (4.0)	0.087
**ANTIBIOTIC RESISTANCE GENES**						
*mecA*	3 (6.3)	54 (25.5)	39 (18.2)	16 (24.2)	44 (44.0)	<**0.001**
*blaZ*	43 (89.6)	179 (85.2)	181 (84.6)	57 (86.4)	90 (90.9)	0.530
*blaI*	43 (89.6)	177 (85.1)	191 (91.0)	57 (86.4)	91 (91.0)	0.341
*blaR*	42 (87.5)	175 (83.3)	177 (84.3)	58 (87.9)	88 (88.9)	0.653
*erm(C)*	2 (4.2)	12 (9.1)	7 (3.3)	9 (13.6)	10 (10.0)	0.018
*msr(A)*	1 (2.1)	29 (13.7)	22 (10.3)	11 (16.7)	34 (34.0)	<**0.001**
*mphC*	1 (2.1)	22 (18.0)	21 (10.0)	8 (17.8)	32 (33.3)	<**0.001**
*aadD*	0 (0.0)	29 (13.7)	20 (9.3)	13 (19.7)	32 (32.6)	<**0.001**
*aphA3*	1 (2.1)	26 (12.3)	12 (5.6)	13 (19.7)	26 (26.3)	<**0.001**
*sat*	1 (2.1)	23 (11.0)	10 (4.7)	12 (18.5)	26 (26.0)	<**0.001**
*mupR*	0 (0.0)	10 (5.4)	10 (4.7)	3 (4.5)	8 (8.0)	0.154
*fosB*	34 (70.8)	159 (75.7)	153 (72.2)	55 (84.6)	75 (75.0)	0.295
**VIRULENCE GENES**						
*tst1*	11 (22.9)	34 (16.1)	43 (20.4)	9 (13.6)	11 (11.0)	0.181
*sea*	18 (37.5)	65 (31.4)	46 (21.5)	20 (30.8)	15 (15.2)	0.003
*sec*	2 (4.2)	18 (8.5)	11 (5.1)	3 (4.5)	4 (4.0)	0.432
*sed*	10 (20.8)	8 (4.1)	23 (11.4)	0 (0.0)	6 (6.6)	<**0.001**
*seg*	34 (70.8)	158 (75.6)	153 (74.3)	45 (68.2)	81 (81.0)	0.391
*sei*	34 (70.8)	134 (72.8)	147 (68.7)	44 (67.7)	80 (81.6)	0.151
*sel*	2 (4.2)	17 (8.1)	11 (5.1)	3 (4.5)	4 (4.0)	0.533
*selm*	36 (75.0)	136 (73.9)	154 (72.6)	45 (68.2)	84 (84.0)	0.137
*seln*	34 (70.8)	139 (75.5)	149 (70.3)	45 (68.2)	81 (81.0)	0.223
*selo*	27 (56.3)	125 (700.6)	144 (68.9)	42 (64.6)	79 (79.0)	0.058
*egc*	35 (72.9)	168 (79.2)	165 (77.8)	45 (68.2)	83 (83.0)	0.215
*selu*	34 (70.8)	164 (77.7)	150 (70.4)	45 (68.2)	83 (83.0)	0.067
*lukS*	44 (91.7)	183 (87.6)	178 (86.4)	63 (98.4)	88 (93.6)	0.011
*hlgA*	47 (97.9)	196 (94.2)	204 (95.8)	61 (93.8)	97 (98.0)	0.448
*lukD*	24 (50.0)	126 (59.4)	120 (56.1)	45 (68.2)	66 (66.0)	0.152
*lukE*	23 (47.9)	117 (55.7)	111 (52.9)	39 (60.0)	67 (67.0)	0.110
*lukX*	41 (85.4)	190 (93.1)	191 (90.5)	63 (95.5)	92 (92.9)	0.325
*hla*	40 (83.3)	187 (88.6)	187 (93.5)	64 (97.0)	97 (97.0)	0.006
*un-disr hlb^α^*	9 (18.8)	87 (41.0)	54 (26.6)	8 (12.1)	15 (15.2)	<**0.001**
*sak*	39 (81.3)	136 (74.3)	166 (79.8)	53 (80.3)	77 (77.0)	0.669
*chp*	33 (68.8)	147 (71.7)	148 (75.1)	5 (75.8)	70 (70.7)	0.814
*scn*	43 (89.6)	181 (85.8)	179 (86.1)	60 (90.9)	81 (81.8)	0.497
*aur*	44 (91.7)	123 (95.3)	199 (95.2)	63 (95.5)	91 (91.9)	0.686
*splA*	24 (50.0)	126 (59.4)	119 (56.4)	45 (68.2)	68 (68.7)	0.084
*splB*	24 (50.0)	122 (57.5)	120 (56.3)	46 (69.7)	69 (69.0)	0.044
*splE*	20 (41.7)	89 (42.6)	103 (48.1)	33 (50.0)	25 (25.0)	**0.001**
**CAPSULE-ASSOCIATED GENES**						
*cap 5*	21 (43.8)	118 (55.7)	109 (50.9)	38 (57.6)	68 (68.0)	0.026
*cap 8*	28 (58.3)	96 (45.3)	105 (49.3)	28 (42.4)	32 (32.0)	0.016
**MSCRAMM GENES**						
*bbp*	45 (93.8)	183 (86.3)	192 (89.7)	57 (86.4)	93 (93.0)	0.273
*cna*	26 (54.2)	79 (38.2)	100 (48.5)	20 (30.8)	32 (32.7)	0.006
*ebh*	45 (93.8)	194 (91.9)	190 (89.2)	64 (97.0)	90 (90.0)	0.257
*fib*	28 (58.3)	132 (62.3)	126 (59.2)	51 (77.3)	71 (71.0)	0.030
*sasG*	23 47.9	127 (59.9)	130 (60.7)	41 (63.1)	70 (70.0)	0.130
*vwb*	44 (91.7)	197 (92.9)	212 (99.1)	63 (96.9)	99 (99.0)	0.002
*isaB*	48 (100.0)	185 (88.5)	111 (62.7)	66 (100)	74 (76.3)	<**0.001**

*Multiple comparison*.

*Unknown bacteremia source (169, 20.3%) and other bacteremia sources including respiratory, abdominal and urinary tract infections (24, 2.9%) were not included in this analysis*.

*Ambiguous results from DNA arrays were considered as missing values for further analysis. Variables with an unacceptable proportion of missing values (>30%) were excluded from analysis*.

* *Only the major clones are shown*.

a *P-values are calculated for each gene with a two-tailed chi-squared or Fisher's exact test, as appropriate. The Bonferroni correction was applied (significant p-value < 0.001). Statistically significant results are highlighted in bold*.

CRB, catheter-related bacteremia; CC, clonal complex;

α *undisrupted hlb; MSCRAMM, microbial surface components recognizing adhesive matrix molecules*.

**Table 4B:** Pairwise comparison of clonal complexes, *agr* types and virulence genes according to source of infection.

**Variable**	**Colonization** ***N*** = **48**			**CRB** ***N*** = **212**			**Endocarditis** ***N*** = **214**			**SSTI** ***N*** = **66**			**Osteoarticular** ***N*** = **100**		
	**YES**	**NO**	***P*-value^a^**	**YES**	**NO**	***P*-value^a^**	**YES**	**NO**	***P*-value^a^**	**YES**	**NO**	***P*-value^a^**	**YES**	**NO**	***P*-value^a^**
CC^*^															
5	9 (19.1)	190 (31.2)	0.117	65 (31.3)	134 (29.9)	0.798	47 (22.0)	152 (34.4)	0.002	19 (29.7)	180 (30.4)	1.000	51 (51.5)	148 (26.6)	< 0.001
8	2 (4.3)	46 (7.6)	0.584	14 (6.7)	34 (7.6)	0.817	22 (10.3)	26 (5.9)	0.062	5 (7.8)	43 (7.3)	1.000	3 (3.0)	45 (8.1)	0.092
15	3 (6.4)	44 (93.6)	0.788	15 (7.2)	193 (92.8)	0.621	20 (9.3)	194 (32.2)	0.568	10 (15.6)	54 (84.4)	0.043	4 (4.0)	95 (96.0)	0.114
22	2 (4.3)	45 (95.7)	0.760	15 (7.2)	193 (92.8)	0.685	16 (7.5)	198 (32.2)	0.541	1 (1.6)	63 (98.4)	0.110	7 (7.1)	92 (92.9)	0.943
30	11 (23.4)	110 (18.1)	0.475	44 (21.2)	77 (17.2)	0.267	41 (19.2)	80 (18.1)	0.825	11 (17.2)	110 (18.6)	0.918	11 (11.1)	110 (19.7)	0.057
45	4 (8.5)	43 (91.5)	1.000	19 (9.1)	189 (90.9)	0.674	19 (8.9)	195 (91.1)	0.789	3 (4.7)	61 (95.3)	0.346	6 (6.1)	93 (93.9)	0.513
***agr*** **TYPES**															
*agrI*	17 (36.2)	30 (63.8)	0.934	72 (34.1)	39 (65.9)	0.942	81 (38.6)	129 (61.4)	0.162	18 (28.1)	46 (71.9)	0.317	28 (28.3)	71 (71.7)	0.190
*agrII*	14 (29.8)	263 (43.3)	0.098	90 (42.7)	187 (42.2)	0.982	77 (36.7)	200 (45.0)	0.052	31 (48.4)	246 (41.7)	0.366	55 (55.6)	222 (40.0)	0.006
*agrIII*	12 (25.5)	35 (74.5)	0.483	47 (22.3)	164 (77.7)	0.498	46 (21.9)	164 (78.1)	0.608	14 (21.9)	50 (78.9)	0.900	12 (12.1)	87 (87.9)	0.035
*agrIV*	4 (8.5)	13 (2.1)	0.028	2 (0.9)	15 (3.4)	0.071	6 (2.8)	11 (2.5)	0.983	1 (1.6)	16 (2.7)	1.000	4 (4.0)	13 (2.3)	0.307
**ANTIBIOTIC RESISTANCE GENES**															
*mecA*	3 (6.3)	162 (26.3)	0.001	54 (25.5)	111 (24.6)	0.875	39 (18.2)	126 (28.0)	0.009	16 (24.2)	149 (24.9)	1.000	44 (44.0)	121 (21.5)	< 0.001
*erm (C)*	2 (4.2)	39 (7.3)	0.564	12 (9.1)	29 (6.4)	0.391	7 (3.3)	34 (9.2)	0.011	9 (13.6)	32 (6.2)	0.049	10 (10.0)	31 (6.4)	0.289
*msr (A)*	1 (2.1)	102 (16.6)	0.003	29 (13.7)	74 (16.4)	0.444	22 (10.3)	81 (18.1)	0.013	11 (16.7)	92 (15.4)	0.934	34 (34.0)	69 (12.3)	< 0.001
*mphC*	1 (2.1)	86 (17.9)	0.002	22 (18.0)	65 (16.0)	0.689	21 (10.0)	66 (20.6)	0.002	8 (17.8)	79 (16.3)	0.967	32 (33.3)	55 (12.7)	< 0.001
*aadD*	0 (0.0)	101 (16.4)	0.001	29 (13.7)	72 (16.0)	0.517	20 (9.3)	81 (18.0)	0.005	13 (19.7)	88 (14.7)	0.377	32 (32.6)	69 (12.3)	< 0.001
*aphA3*	1 (2.1)	81 (13.2)	0.021	26 (12.3)	56 (12.4)	1.000	12 (5.6)	70 (15.6)	< 0.001	13 (19.7)	69 (11.6)	0.089	26 (26.3)	56 (9.9)	< 0.001
*sat*	1 (2.1)	76 (12.4)	0.033	23 (11.0)	54 (12.0)	0.810	10 (4.7)	67 (15.1)	< 0.001	12 (18.5)	65 (10.9)	0.112	26 (26.0)	51 (9.1)	< 0.001
**VIRULENCE GENES**															
*sea*	18 (37.5)	155 (25.5)	0.098	65 (31.4)	108 (24.0)	0.057	46 (21.5)	127 (28.7)	0.063	20 (30.8)	153 (25.8)	0.479	15 (15.2)	158 (28.3)	0.009
*sed*	10 (20.8)	39 (6.7)	0.001	8 (4.1)	41 (9.6)	0.027	23 (11.4)	26 (6.1)	0.031	0 (0.0)	49 (8.7)	0.006	6 (6.6)	43 (8.0)	0.793
*selo*	27 (56.3)	408 (71.0)	0.049	125 (70.6)	310 (69.5)	0.860	144 (68.9)	291 (70.3)	0.791	42 (64.6)	393 (70.4)	0.410	79 (79.0)	356 (68.1)	0.039
*selu*	34 (70.8)	460 (74.9)	0.650	164 (77.7)	330 (73.2)	0.246	150 (70.4)	344 (76.6)	0.106	45 (68.2)	449 (75.3)	0.264	83 (83.0)	411 (73.1)	0.049
*lukS*	44 (91.7)	532 (89.1)	0.808	183 (87.6)	393 (90.1)	0.392	178 (86.4)	398 (90.7)	0.136	63 (98.4)	513 (88.3)	0.009	88 (93.6)	488 (88.6)	0.199
*hla*	40 (83.3)	559 (93.0)	0.032	187 (88.6)	412 (94.1)	0.023	187 (93.5)	412 (91.8)	0.543	64 (97.0)	535 (91.8)	0.218	97 (97.0)	502 (91.4)	0.086
*un-disr hlb^α^*	9 (18.8)	168 (27.8)	0.234	87 (41.0)	90 (20.5)	< 0.001	54 (26.6)	123 (27.4)	0.908	8 (12.1)	169 (28.8)	0.006	15 (15.2)	162 (29.3)	0.005
*splA*	24 (50.0)	374 (61.1)	0.173	126 (59.4)	272 (60.7)	0.819	119 (56.4)	279 (62.1)	0.187	45 (68.2)	353 (59.4)	0.213	68 (68.7)	330 (58.8)	0.082
*splB*	24 (50.0)	372 (60.5)	0.203	122 (57.5)	274 (60.8)	0.484	120 (56.3)	276 (61.3)	0.254	46 (69.7)	350 (58.6)	0.108	69 (69.0)	327 (58.1)	0.052
*splE*	20 (41.7)	262 (42.7)	1.000	89 (42.6)	193 (42.7)	1.000	103 (48.1)	179 (40.0)	0.060	33 (50.0)	249 (41.8)	0.255	25 (25.0)	257 (45.8)	< 0.001
**CAPSULE-ASSOCIATED GENES**															
*cap 8*	28 (58.3)	272 (44.2)	0.082	96 (45.3)	204 (45.2)	1.000	105 (49.3)	195 (43.3)	0.175	28 (42.4)	272 (45.6)	0.722	32 (32.0)	268 (47.6)	0.005
**MSCRAMM GENES**															
*cna*	26 (54.2)	241 (40.2)	0.081	79 (38.2)	188 (42.6)	0.321	100 (48.5)	167 (37.8)	0.012	20 (30.8)	247 (42.4)	0.095	32 (32.7)	235 (42.7)	0.079
*fib*	28 (58.3)	396 (64.4)	0.493	132 (62.3)	292 (64.7)	0.593	126 (59.2)	298 (66.2)	0.092	51 (77.3)	373 (62.5)	0.025	71 (71.0)	353 (62.7)	0.139
*vwb*	44 (91.7)	592 (96.3)	0.124	197 (92.9)	439 (97.3)	0.013	212 (99.1)	424 (94.4)	0.003	63 (96.9)	573 (95.8)	1.000	99 (99.0)	537 (95.4)	0.104
*isaB*	48 (100)	458 (80.1)	0.001	185 (88.5)	321 (78.1)	0.002	111 (62.7)	395 (89.2)	< 0.001	66 (100)	440 (79.4)	< 0.001	74 (76.3)	432 (82.6)	0.183

* *Only the major clones are shown*.

SAB, Staphylococcus aureus bacteremia; CRB, catheter-related bacteremia; CC, clonal complex;

α *undisrupted hlb; MSCRAMM, microbial surface components recognizing adhesive matrix molecules*.

a *P-values are calculated for each gene with a two-tailed chi-squared or Fisher's exact test, as appropriate. The Bonferroni correction was applied (significant p-value < 0.001)*.

A comparison of colonizing vs. SAB strains revealed that the *sed* gene was most frequently found in colonizing strains (20.8 vs. 6.7%, *p* < 0.001; Table 4B). This gene also had greater representation among endocarditis strains (11.4 vs. 6.1%, *p*: 0.031). In addition, the undisrupted *hlb* gene mostly presented in CRB strains (41.0 vs. 20.5%, *p* < 0.001; Table 4B). The presence of MSCRAMM genes, such as *cna, fib, vwb* and *isaB* also varied significantly according to source of SAB. While *cna* and *vwb* genes had greater representation among strains from an endocarditis source (48.5 vs. 37.8%, *p*: 0.012 and 99.1 vs. 94.4%, *p*: 0.003, respectively), there was significant detection of *fib* genes (77.3 vs. 62.5%, *p*: 0.025) in SSTI sources. Finally, the *isaB* gene was more commonly found among CRB strains (88.5 vs. 78.1%, *p*: 0.002) and in the SSTI bacteremia group (100 vs. 79.4%, *p* < 0.001; Table 4B).

Various regression models were performed in order to measure the role of pathogen-related molecular markers (CC, *agr* type and virulence genes) adjusted for different sources of bacteremia and colonization (Table 5). All adjusted models in multivariate analysis showed that these variables were the presence of *agrIV* type and *sed* gene for colonizing strains; the presence of *sea*, undisrupted *hlb* and *isaB* genes for CRB; *sed, splE* and *fib* genes for an endocarditis source; undisrupted *hlb* for the SSTI group; and finally, CC5, *msrA* resistance gene and *hla* gene with respect to bacteremia from an osteoarticular source (Table 5).

**Table 5:** Multivariate analysis according to different sources of SAB and colonization.

	**Colonization**		**CRB**		**Endocarditis**		**SSTI**		**Osteoarticular**	

**Variable**	**aOR (95% CI)**	***P*****-value**	**aOR (95% CI)**	***P*****-value**	**aOR (95% CI)**	***P*****-value**	**aOR (95% CI)**	***P-*****value**	**aOR (95% CI)**	***P*****-value**
*CC5*									2.05 (1.18–3.64)	0.011
*agrIV*	4.11 (1.24–13.66)	0.021								
*mecA*	0.18 (0.05–0.060)	0.005			–	–				
*erm(C)*					–	–				
*msr(A)*									2.55 (1.37–4.75)	0.003
*aadD*									–	–
*sat*					0.41 (0.19–0.87)	0.021				
*sea*			2.06(1.34–3.15)	0.001	0.57 (0.34–0.96)	0.035			0.39(0.21–0.72)	0.003
*sed*	5.22 (1.21–12.35)	< 0.001	0.24(0.09–0.64)	0.004	2.79 (1.33–5.85)	0.007	–	–		
*selu*									–	–
*lukS*										
*hla*	0.33 (0.14–0.78)	0.012	0.42 (0.22–0.79)	0.008					10.01 (1.34–76.01)	0.025
*un–disr hlb^α^*			3.72 (2.42–5.71)	< 0.001			2.5 (1.15–5.46)	0.029	0.30 (0.16–0.56)	< 0.001
*splE*					1.56 (1.01–2.43)	0.046				
*cap 8*									–	–
*cna*	–	–								
*fib*	–	–			3.42 (1.63–6.44)	0.001				
*vwb*			0.35 (0.15–0.80)	0.013						
*isaB*			2.62 (1.50–4.59)	0.001	0.05 (0.02–0.10)	< 0.001	–	–		

*Various multivariate models were explored that included different numbers of variables according to the number of events by bacteremia source*.

*–: variables included in the initial model of multivariate analysis then discarded in a backward stepwise process. Only variables consistently retained in exploratory models are shown*.

CRB, catheter-related bacteremia; aOR, adjusted Odds Ratio; 95% CI, 95% confidence intervals;

α *undisrupted hlb*.

## Discussion

The present study describes and gives a global epidemiological overview of the molecular epidemiology of a large collection of *S. aureus* strains focused on bloodstream infections in Spain over a 15-years period. In this scenario, our study provides important findings regarding the distribution of clonality and virulence genes and their association with specific sources of SAB.

Our results revealed high clonal diversity among SAB strains, although the most prevalent CCs were CC5, CC30, CC45, CC8, CC15, and CC22, which together represented 80% of all cases. Additionally, substantial differences were found between strains causing MRSA and MSSA bacteremia, which indicated that MSSA strains were much more genetically diverse than their MRSA counterparts, which is consistent with studies developed in Europe (Aamot et al., 2012; Grundmann et al., 2014) and the USA (Miko et al., 2013; Park et al., 2017). The most common clone among MSSA strains was CC30, followed by CC45 and CC15, whereas among MRSA strains, there was a significant representation of CC5 in more than 75% of strains. Similar results have been reported in Latin America (Arias et al., 2017) and Germany (Schaumburg et al., 2012), where the CC5-MRSA clone was the most prevalent in the setting of bloodstream infections. Furthermore, in our collection, CC5 was found to be significantly associated with HA acquisition and the adult population, a finding which lends support to the interest aimed at investigating the pathogenic and molecular characteristics of CC5 and those factors that enhance its spread.

Several studies have suggested that while the *agrI* type is the most common one among clinical isolates (van Leeuwen et al., 2000; Moore and Lindsay, 2001), others (Sakoulas et al., 2003) have determined that more than half of clinical MRSA bloodstream isolates belong to *agr* group *II*. In our collection, *agrII* was also associated with MRSA, which may explain the higher percentage of *agrII* found in the nosocomial setting and among adults, in whom the prevalence of MRSA was higher. By contrast, *agrI* was related to MSSA strains and CA acquisition. Interestingly, a statistically significant association was also found between *agrII* and *agrIII* and adult and child populations, respectively. These associations are probably due to the correlation between *agr* type and CC, since CC5 (*agrII*) was the majority clone among adults and CC30 (*agrIII*) among children.

To date, different studies have explored the association between bacterial genotype, especially *S. aureus* virulence genes, and various clinical syndromes (Gillet et al., 2002; Jarraud et al., 2002; Peacock et al., 2002). This study focuses specifically on bacteremia. Our collection included *S. aureus* strains from the most common primary clinical sources of infection: CRB, SSTI, osteoarticular infection and endocarditis, as well as nasal carriage strains. We found no major differences between colonizing and bacteremia-producing strains of *S. aureus*, which supports the fact that most strains of *S. aureus* are capable of causing bacteremia. Nevertheless, and in accordance with other studies (Fowler et al., 2007; Giulieri et al., 2016), we identified specific clonal backgrounds and various molecular markers that have been associated with bloodstream infections and certain sources of bacteremia in particular. In this regard, our findings showed that CC5 in addition to *hla* and *msrA* genes were more frequently present in strains causing osteoarticular bacteremia. The association of the *hla* gene, present in most *S. aureus* strains, with different types of infection has already been reported (Stulik et al., 2014; Sharma-Kuinkel et al., 2015). Further studies are needed to elucidate the role of this important virulence factor in the pathogenesis of bacteremia. With respect to the adhesin genes (MSCRAMMs), which play an essential role in the pathogenesis of intravascular, osteoarticular and device-associated *S. aureus* infections (Foster et al., 2014), our study revealed an association between the *fib* and *isaB* genes and endocarditis and CRB sources, respectively. Other adhesins like *clfA/B, fnbA/B*, and *cna* and their linkage with bacteremia, endocarditis and CRB, have also been reported (Giulieri et al., 2016; San-Juan et al., 2017).

Another finding of note in this study was the presence of the undisrupted β-hemolysin (*undisrupted hlb*) which was significantly related to sources, such as CRB and SSTI. Different studies have demonstrated its contribution to SSTI (Hedström and Malmqvist, 1982; Lebughe et al., 2017) and biofilm-related infections (Salgado-Pabón et al., 2014). Although β-toxin is encoded in *S. aureus*, most strains are reported not to secrete β-toxin because the bacteriophage (ϕSa3) inserts into the *hlb* gene (Winkler et al., 1965; Coleman et al., 1991), inactivating it in the majority of *S. aureus* strains recovered from humans. Moreover, the ϕSa3 bacteriophage encodes the immune evasion cluster (IEC) *sak-chip-scn* (Coleman et al., 1989; de Haas et al., 2004). Coinciding with other studies (Pantucek et al., 2004; Van Wamel et al., 2006), these genes were relatively abundant in our collection, ranging between 73% (*sak*) and 87% (*scn*). Interestingly, the absence of the intact *hlb* gene (or which amounts to the same thing, the presence of *hlb* truncated by the IEC-carrying ϕSa3 phage) was significantly associated with an osteoarticular source. This intriguing association should be investigated further since other studies have reported the association between these phage-integrated genes and less severe staphylococcal infections (Jin et al., 2003).

This study presents several limitations that should be mentioned. First, the heterogeneity and non-continuity of the SAB collection (geographical origin, time points and hosts) precluded us from adjusting for these variables in multivariate analysis. Moreover, the proportion of colonization strains was small in comparison with the number of SAB strains. The results therefore should be interpreted with caution. At the same time, our study includes a large number of *S. aureus* strains causing bacteremia, with relevant information on place of acquisition, methicillin resistance and source of infection. Second, the lack of clinical data regarding the outcome of the bacteremic episodes makes it impossible to make inferences about the prognostic importance of the molecular factors. Other studies evaluating associations between bacterial genotype and virulence have led to conflicting results (Day et al., 2001, 2002; Feil et al., 2003; Melles et al., 2004), due in part to the heterogeneous nature of the *S. aureus* infections included, as well as the absence of a large, well-characterized collection of isolates. Third, our study methodology was based on DNA microarrays, which should be noted in the case of the *hla* gene. Despite the fact that *hla* is present in virtually all *S. aureus* strains, some studies, such as Sharma-kuinkel et al. (JCM) have reported up to 12 different variants of *hla*. In our study, the *hla* gene was detected in 92.2% of strains. We think that the low frequency of this gene observed in our collection may have been due to the DNA microarray technology, which may underestimate the presence of certain minority *hla* variants due to lack of sensitivity. Whole genome sequencing may be a more effective genotypic characterization approach for detecting different genetic variants that may not be detected by hybridization procedures, although previous studies have shown good agreement between the genotypic results obtained using a DNA array-based methodology and those using high-throughput sequencing (Strauß et al., 2016). Finally, we did not perform gene expression studies, which would be key to determining whether a particular gene or set of genes was responsible for the specific pathogenic behavior observed in SAB from particular clinical sources. Nevertheless, our findings offer a valuable starting point for further research insights into intrinsic pathogenic mechanisms involved in the development of SAB.

In conclusion, the current study suggests a potential association between *S. aureus* genotype and acquisition, methicillin resistance and bloodstream infection sources. The results of this study reinforce the view that SAB continues to represent a major clinical challenge. Thus, a better understanding of *S. aureus* epidemiology and pathogenesis is crucial to the detection of prognostic biomarkers as well as to the development of potential therapeutic targets aimed at improving patient outcomes.

## Author contributions

DP-M, EV, and FC conceived and designed the experiments. EV, CG-G, ERG, NL, NF-H, RS, and IM-G collected the isolates. Funding was obtained by FC and BA. Experiments were performed by EV, CG-G, and IM-G. The data were analyzed by EV and DP-M. EV and DP-M prepared the manuscript draft. All authors agreed to be accountable for all aspects of the work. EV, DP-M, and FC contributed in giving final approval of the version to be published. All authors reviewed and approved the final manuscript.

### Conflict of interest statement

The authors declare that the research was conducted in the absence of any commercial or financial relationships that could be construed as a potential conflict of interest.
